# Smooth velocity fields for tracking climate change

**DOI:** 10.1038/s41598-022-07056-z

**Published:** 2022-02-22

**Authors:** Iaroslav Gaponenko, Guillaume Rohat, Stéphane Goyette, Patrycja Paruch, Jérôme Kasparian

**Affiliations:** 1 DQMP, University of Geneva, Quai Ansermet 24, 1211 Geneva 4, Switzerland; 2 Institute for Environmental Sciences, University of Geneva, bd Carl Vogt 66, 1211 Geneva 4, Switzerland; 3 Group of Applied Physics, University of Geneva, Chemin de Pinchat 22, 1211 Geneva 4, Switzerland

**Keywords:** Climate change, Projection and prediction, Scientific data

## Abstract

Describing the spatial velocity of climate change is essential to assessing the challenge of natural and human systems to follow its pace by adapting or migrating sufficiently fast. We propose a fully-determined approach, “MATCH”, to calculate a realistic and continuous velocity field of any climate parameter, without the need for *ad hoc* assumptions. We apply this approach to the displacement of isotherms predicted by global and regional climate models between 1950 and 2100 under the IPCC-AR5 RCP 8.5 emission scenario, and show that it provides detailed velocity patterns especially at the regional scale. This method thus favors comparisons between models as well as the analysis of regional or local features. Furthermore, the trajectories obtained using the MATCH approach are less sensitive to inter-annual fluctuations and therefore allow us to introduce a trajectory regularity index, offering a quantitative perspective on the discussion of climate sinks and sources.

## Introduction

Global climate change already has observable effects on the environment and on humankind, with drastic changes at all scales impacting and threatening many ecological niches and human activities^1^. To understand and address these present and future challenges, stakeholders, decision makers, and other practitioners need medium- to long-term projections to develop and implement mitigation as well as adaptation strategies^2^. While such data are provided by regional and global climate models (RCMs and GCMs), displaying them to optimize their intelligibility especially for non-specialists is still a challenge in itself.

Adaptation to climate change strongly depends on its pace. Ecosystems^3,4^ as well as human systems (forestry^5^, agriculture^6,7^, or urban planning^8,9^)^10–12^ have limited migration and evolution capabilities. The adaptation of human activities and ecological mobility (*e*.*g*., species or biome shifts) must therefore be faster than the velocity of climate change to avoid extinction.

It is thus of utmost importance to adequately and accurately assess the velocity of climate change in space and time. However, both global and regional climate models simulate the time evolution of the climate system on a fixed grid, and their output in terms of temporal evolution needs to be adapted and translated into a spatial evolution, *i*.*e*., a shifting velocity of the considered climate zones. A first step in this direction was the introduction of a *velocity index*^3^ estimating the spatial velocity *v* of climate change (especially focusing on temperature). It is defined as the ratio of the local rate of change (temporal derivative, sometimes termed “temporal gradient”) of temperature to the magnitude of its spatial gradient:

1 $$\begin{aligned} v = \frac{\partial T}{||\nabla T|| \partial t} \end{aligned}$$

Such local velocity can be calculated for any scalar field $\psi$ (*e*.*g*., temperature, precipitation, *etc*.), and interpreted as the displacement speed of the corresponding isopleths. However, we recently emphasized that this approach would only be mathematically correct if the isopleths would move down the gradient of $\psi$, parallel to each other, and without deformation^13^. This would typically be the case if they were to uniformly shift polewards, following geodesic parallels. While this is expected in the case of a homogeneous planet (*e*.*g*., an aquaplanet), such an assumption is more than questionable for today’s Earth, where land-ocean inhomogeneities, coasts, altitude differences (*i*.*e*. topography), or marine currents carrying heat flows obviously bend the isotherms and steer them along non-trivial paths, affecting their displacement and deforming their shape.

**Figure 1:**
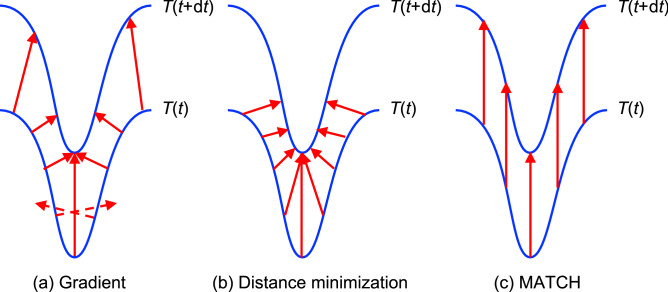
Schematic illustration of the challenge encountered when defining the climate shifting velocity field in regions where curved isotherms shift northwards. We consider the displacement of isotherm *T* from time *t* to time $t+\text {d}t$ (blue lines). (**a**) Gradient-based velocity. The shifting (red arrows) occurs along the gradient, i.e., perpendicular to the isotherms. The velocity field exhibits shear and artificial convergence, particularly marked in the case of the dashed arrows, that furthermore do not reach the isotherm of time $t + \text {d}t$. (**b**) Velocity field minimizing the displacement. Shear, and local bunching and loosening is also unavoidable in curved regions of the isotherms. (**c**) MATCH approach, yielding a velocity field as uniform and as little sheared as possible.

The limitations of the above assumption are exemplified in Fig. 1, which considers a typical situation of curved isotherms (blue curves) shifting northward. Figure 1a illustrates the analysis of this shift (red arrows) as a deformation following the gradient, i.e., perpendicular to the initial isotherm. Several issues arise. First, a curved isotherm results in a sheared velocity field. Second, the gradient approach is numerically unstable as it artificially leads to the local convergence of the velocity field (See bottom part of the scheme), leading to artificial sinks if the temporal step is too long. The dashed arrows illustrate the extreme case of an artificial divergence of the gradient-based velocity. Furthermore, then do not point to the isotherm of time $t + \text {d}t$. Numerical divergence to infinite velocities also occurs where the gradient vanishes to zero, although usual software packages discard such points. Such discontinuity is intrinsic to the gradient-based approach. According to the hedgehog (“Hairy ball”) theorem^14^, the gradient cancels out in at least one location on a spherical earth. At these locations, the velocity index as calculated through Equation (1)^3^ diverges to infinity.

Although a direct application to biome migration or the comparison with observational data are beyond the scope of the present work, it should be noted that the seemingly natural assumption that, in the absence of obstacles, species or biomes would minimize their migration effort by travelling along the gradient is far from obvious. First, as illustrated in Fig. 1b, trajectories minimizing the displacement have no reason, in general, to follow the gradient. Furthermore, considering species or biomes with non-zero existence areas, the convergence (divergence) regions artificially brought by the gradient method would yield to over- (under-) crowded regions, likely influencing the trajectories to a more even occupation of the available niches offering adequate climate, therefore resulting in a more regular velocity field. Indeed, substantial anisotropies and deviations from a poleward migration not imposed by barriers have recently been observed in numerous species^15^.

In fact, in the situation of Fig. 1, the uniform velocity field of Panel (c), describing the evolution as a northward shift of the isotherm rather than a complex deformation implying local shrinkings and stretchings, can be seen as visually more appealing.

Here, we propose a way to build such velocity field, and show that the limitations of the gradient method can be overcome by effectively calculating a continuous field of climate change velocity for any scalar field issued simulated by regional or global climate models, providing a more detailed, smoother velocity field with less vorticity and sensitivity to noise such as inter-annual fluctuations. We show, especially at the regional scale, that this approach helps the effects of climate change to be addressed and adaptation issues to be discussed. Finally, based on the local properties of the velocity field, we define a trajectory regularity index which offers a quantitative perspective on the discussion of climate sinks and sources.

The coexistence of multiple mathematical solutions to the isopleth velocity field problem arises from the fact that the local shifting velocity is a two-dimensional vector field featuring both magnitude and direction, or equivalently, *x* (zonal, *i*.*e*. pointing east) and *y* (meridional, *i*.*e*. pointing north) components, while the equation linking it to the rate of change and the gradient of the field $\psi$ is scalar:

2 $$\begin{aligned} \frac{\partial \psi }{\partial t} = \vec {\nabla } \psi \cdot \vec {v} \end{aligned}$$

*i*.*e*., one-dimensional, and therefore only provides incomplete information about the system. Determining the velocity vector field $\vec {v}$ thus requires closing the problem. This implies a need to complement Eq. (2) with a second equation. Loarie et al.^3^ fill this gap by assuming that isopleths shift along the gradient. While simple and seemingly intuitive, this *ad hoc* assumption induces the biases discussed above.

In contrast, we close the problem of eq. (2) by imposing the minimisation of the velocity field shear, so as to prevent discontinuities. We implement this approach by using an iterative Monte-Carlo-based technique (**M**onte-C**a**rlo I**t**eration/**C**onvergence Met**h**od, hereafter referred to as MATCH, See Methods for details). We illustrate this continuity-based approach at both the global and regional scales (using simulated screen-level air temperature and pressure outputs from climate models in the frame of the Representative Concentration Pathway RCP8.5 emission scenario of the IPCC-AR5^16^.

The resulting climate trajectory maps highlight local features constraining the spatial drift of climate variables, such as the orography or coastal regions, with a remarkable contrast between land and ocean behaviors. Our approach therefore provides a rich pedagogic tool to display climate evolution dynamics. The resulting velocity-field maps are more relevant to investigating biome shift, as discontinuities in these would unrealistically exaggerate occurrences of convergence, and therefore spatial crowding of biomes or species in limited areas. Such crowding would result in interferences, challenging the migration of multiple ecosystems into the same region in spite of their individual capability to keep up with the pace of climate change.

Moreover, the continuity-based approach maximises the mathematical stability of the shifting velocity against local singularities, or inter-annual fluctuations. It therefore enables high-resolution (up to yearly) analysis of individual trajectories. The resulting maps open the opportunity to assess the migration ability of species or biotopes at finer temporal and spatial scales, accounting for both transient accelerations of the pace of climate change, and detours in the trajectories which migrating species or biomes would tend to follow. Such features are, *e*.*g*., at the root of the faster effective shifting velocities of marine species as compared to terrestrial ones^17^. Finally, the proposed MATCH approach allows us to characterize the stability of trajectories so as to better understand climate displacement dynamics, by defining several metrics based on the observed or predicted trajectory fields.

## Results

### Climate shifting velocity fields

#### Global scale

**Figure 2:**
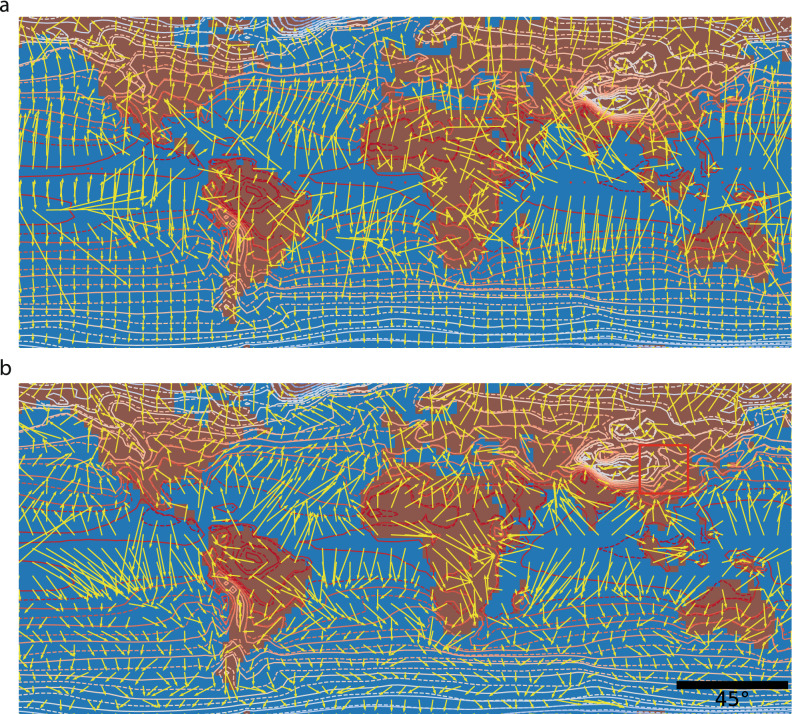
Shifting velocity field of isotherms between periods 2006–2036 and 2070–2100. The isotherms are taken from the CCCma CanESM2 model for the RCP 8.5 scenario. The displacement is calculated with (**a**) the gradient approach; (**b**) MATCH. The arrows represent isotherm displacement between the two considered periods (70 years apart), and can equivalently be viewed as a velocity. Regions with arrow crossings correspond high vorticity and/or fast shifts. Note that the arrow length does not necessarily correspond to the actual trajectories, since the velocity field deforms with time, bending and accelerating/decelerating trajectories. The red square in panel (**b**) defines the region enlarged in Fig. 7. Red dots in the gradient approximation represent displacement beyond 40 pixels apart (given 2.82° per pixel), which were removed from consideration due to their nonphysical signature. This figure has been produced using the code available in the Additional online information (See “Supplementary Materials”), using the Anaconda distribution of Python 3.6.8, with matplotlib 3.1.1 and numpy 1.14.3, available at https://www.anaconda.com/.

The global shifting velocity fields of surface air temperature and mean sea-level pressure between the periods 2006–2036 and 2071–2100 as simulated with the CCCma CanESM2 GCM^18,19^ for the RCP 8.5 scenario, calculated with both the gradient and the continuity-based MATCH methods, are shown in Fig. 2 for the temperature and in Figure S2 for the pressure data. The representation is an equally spaced grid with the longitude ranging from -170° to 170° (reduced due to computational edge effects) and latitude ranging from − 80° to 80° (reduced due to polar divergence). In both approaches, the isotherm velocity field emerges from the equator and converges to the poles, in line with the overall poleward displacement of the isotherms under climate warming. However, due to the coarse resolution (128x64 pixel grid covering the whole Earth), only the poleward displacement appears in the gradient-based calculation (Fig. 2a). Moreover, the gradient-based velocity field is extremely smooth over the ocean, although divergence artificially appears e.g. north of Australia or in the equatorial region of the Pacific Ocean and the tropical part of the South Atlantic Ocean. In contrast, MATCH (Fig. 2b) yields additional features such as local sources and sinks which clearly appear throughout the map: they are mostly related to topographical features such as coastlines and major mountain chains like in central Africa. Furthermore, eastward or westward displacements clearly appear as consistent patterns^20^, *e*.*g*. over mid-latitude Asia or North America or Amazonia.

Note that, in both cases, the scale chosen for arrow lengths visually leads to arrow crossing, although this does not correspond to trajectory crossing, but rather to consequences of local twisting. Furthermore, in the gradient-based map, some very high velocity arrows appear (*e*.*g*., over Australia, or across the whole South America). They originate from regions where the gradient is close to zero, so that Eq. (1) diverges. The common practice of the gradient method, implemented in the usual software packages, is to discard these data points. However, as they are due to mathematical artifacts intrinsic to the gradient approach, we prefer to display them in order to allow a fair comparison between the two approaches.

#### Regional scale

**Figure 3:**
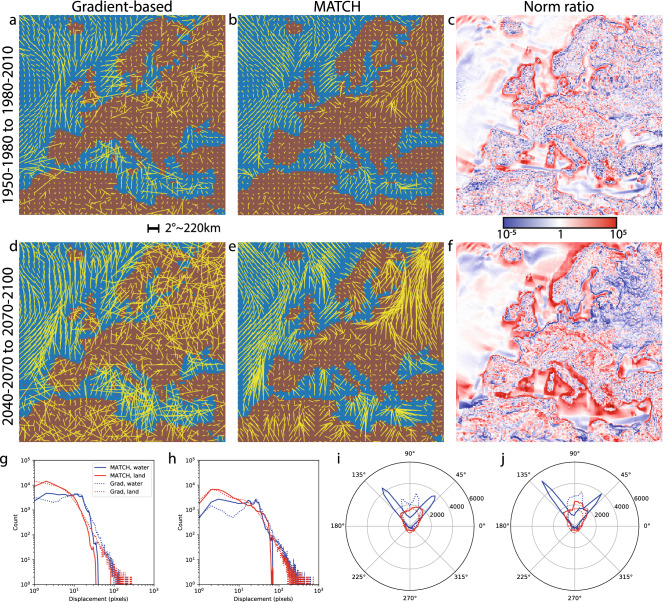
Regional isotherm shifting fields based on the gradient-based method (**a**, **d**) and MATCH (**b**, **e**), for the surface temperature under the RCP8.5 scenario as computed by the RACMO model. We consider the average displacement over thirty-year periods: (**a**–**c**; **g**, **i**) 1950–1980 to 1980–2010 and (**d**–**f**; **h**, **j**) 2040–2070 to 2070–2100. Red dots in the gradient approximation signify displacements beyond 100 pixels (at 0.11° per pixel), which were not displayed to improve the map legibility and owing to their nonphysical nature. Ratio between the norms of the gradient-based and MATCH-based shifting fields (**c**, **f**) . (**g**, **h**) Probability distribution functions using the the gradient (dashed lines) and MATCH (solid lines) based shifts, split over land masses (red) and water areas (blue). (**i**, **j**) Angular distribution of the gradient (dashed lines) and MATCH (solid lines) based, split over land (red) and water areas (blue). This figure has been produced using the code available in the Additional online information (See “Supplementary Materials”), using the Anaconda distribution of Python 3.6.8, with matplotlib 3.1.1 and numpy 1.14.3, available at https://www.anaconda.com/.

Focusing on the Euro-Cordex computational domain (Fig. 3) allows a more detailed analysis of the features discussed in the previous section. MATCH yields smooth velocity fields over most of Europe, as well as Northern Africa and the Mediterranean in the RCP 8.5 scenario. This is especially the case for the second half of the 21st century, where displacements are largest (Panels b,e). In contrast, the gradient-based method displays more erratic shifting directions in this region, which jeopardizes the map legibility (Panels a,d). Such erratic shifts occur already in the period between years 1950–1980 and 1980–2010 (Panels a–c), although the shifting velocity is much slower than that extracted between 2040–2070 and 2070–2100 (Panels d–f). Such unstable shifting directions correspond to the averaging out to the raw poleward shift obtained at the global scale (Fig. 2a).

The MATCH approach (Panels b,e) clearly shows regionally varying behaviours, as well as local features. In particular, one can observe three types of collective behaviour:

- A wide, fast, and relatively homogeneous flow in flat and homogeneous regions, whether over the Atlantic ocean or the North-Eastern European plains.
- Very slow displacements and sinks, either in the central European plains or correlated to topographic variations such as ones caused by local altitude maxima, whether on islands or in mountainous regions like the Atlas, Scandinavia, and to a lesser extent Iberia.
- The attraction of the coastlines, where the temperature contrast (hence, the gradient) between land and sea is well marked, allowing them to act as flow guides.

**Figure 4:**
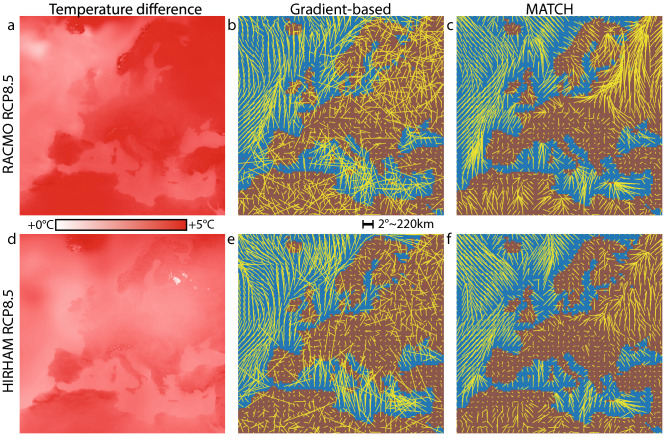
Air temperature change for the RCP 8.5 scenarios between 2040–2070 and 2070–2100, from RACMO (**a**–**c**) and HIRAM (**d**–**f**) models. (**a**,**d**): Temperature change; (**b**,**e**) Gradient-based and (**c**,**f**) MATCH-based shifting velocity maps. Red dots in the gradient approximation signify displacements beyond 100 pixels (at 0.11° per pixel), which were removed from consideration due to their nonphysical nature. This figure has been produced using the code available in the Additional online information (See “Supplementary Materials”), using the Anaconda distribution of Python 3.6.8, with matplotlib 3.1.1 and numpy 1.14.3, available at https://www.anaconda.com/.

The smooth and less vortical MATCH-based velocity maps also visually enhance features from models, favoring direct model comparisons. Figure 4 compares the output of RACMO and HIRAM for the RCP 8.5 scenario between 2040–2070 and 2070–2100, displayed as both the temperature evolution, the gradient-based shifting velocity map, and the continuity-based shifting velocity map. In the temperature difference map, the main difference appears as a 1–2 °C lower temperature increase in HIRAM as compared to RACMO over Central Europe (Panels a,d). The MATCH velocity maps (Panels c,f) show that this difference stems primarily from a much slower and less uniform isotherm drift over North-Eastern Europe, and to a lesser extent from the absence of a sink in Northern France. Due to the noise in the velocity calculation, the gradient-based velocities (Panels b,e) provide less clear conclusions in this regard.

The wide misestimation of the shifting velocities by the gradient method in many situations can be clearly seen by examining the ratio between the outcomes of the two approaches (Fig. 3c,f). Factors beyond 10^5^ are measured, especially in coastal regions as well as at the edge of water masses in the ocean, where the gradient vanishes. Conversely, the ratio can be below 10^−5^, demonstrating large inconsistencies over land masses as well as in specific regions delimiting distinct behaviours.

The probability distribution function for the shifting velocities (Fig. 3g,h) displays a characteristic power-law tail in the case of the gradient method, while it is limited to displacements below 100 pixels (at 0.11° per pixel) over a period of 30 years for the MATCH-based velocities. Additionally, slow displacements are more frequent with the MATCH approach - correlated in fact with much more realistic displacements. These behaviours are observed equally on land and over marine areas.

The angular histograms (Fig. 3i,j) show a similar trend for the two methods. Both approaches produce a general poleward-directed shift over land, as expected from warming in the Northern hemisphere. In marine regions, the angles are spread between NE and NW, with additional texturing due to uniform displacement regions and isotherms gliding along the coasts.

This behavior illustrates that the gradient method cannot provide reliable estimates of shifting directions in regions of small gradient values where the velocity direction becomes mathematically irrelevant. It is therefore highly questionable as a means of identifying source and sink regions^21^, *i*.*e*., regions where new climate emerges, or where pre-existing climatic conditions vanish. This is especially critical for adaptation studies, since sinks are expected to be related to extinction of the corresponding biome^22^, whereas sources can involve the emergence of new ecological niches.

### Isotherm trajectories

As it is relatively immune to noise, the continuity-based MATCH approach enables us to calculate the displacements of isotherms (and more generally, isopleths) over shorter time scales, down to yearly resolution, at which local inhomogeneities and noise play a larger role. The successive displacements of isotherms at the different time steps can be used to stitch together the full trajectory of isotherms starting from specific points, defining the full deformation of their portrait. As the trajectories are generally curved, considering a higher temporal resolution increases the effective displacement velocity as compared to a simple straight line between the initial and final time frames. This can, for instance, lead to more constraints on species or biomes which have to iteratively accommodate to the climate shift along their journeys, and not only jump from the initial to the final locations. Furthermore, as we consider the successive displacements of the same isotherm over time, our trajectories are more accurate than those determined from a static map of velocity averaged over a wide time interval^23^.

As the visualization of a large amount of trajectories poses a perceptional challenge, a trajectory navigator software was developed in order to view the forward and backward trajectories. It is written in python and run by the Anaconda distribution of Python 3.6.8, with matplotlib 3.1.1 and numpy 1.14.3, available at https://www.anaconda.com/. It is freely available at the *Yareta* repository of this work alongside the generated trajectory data files for the model and conditions above^24^. A smoothed animation file depicting a set of trajectories developing as a function of time is also available in the “Supplementary Materials”.

**Figure 5:**
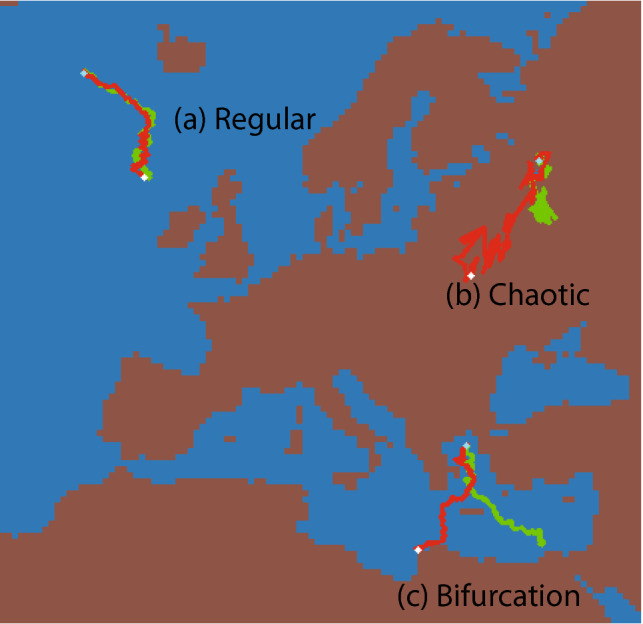
Typical behaviors of the smoothed yearly-resolution forward (green line) and backward (red line) trajectories for the 1950–2100 evolution of temperature for the RCP8.5 scenario, according to the HIRHAM model. (**a**) Regular: the forward and backward trajectories overlay; (**b**) Chaotic: the start and endpoint of the forward-backward trajectories lie somewhat together but both trajectories and endpoints start to diverge; (**c**) Bifurcations: the full trajectories, including the start and endpoints, are radically different. This figure has been produced using the code available in the Additional online information (See “Supplementary Materials”), using the Anaconda distribution of Python 3.6.8, with matplotlib 3.1.1 and numpy 1.14.3, available at https://www.anaconda.com/.

From a visual analysis of the resulting data set, we identified three main types of behaviors across the Euro-Cordex domain (Fig. 5). In regions where the shifting velocity field is close to homogeneous (See Label a in Fig. 5), forward and backward trajectories almost overlap with each other. These *regular* trajectories mostly occur above bodies of water, where there are no orographic effects. Conversely, a *bifurcation* regime is observed in regions of velocity map convergence (sinks) or divergence (sources). In this case, multiple trajectories share a similar destination, with the back trajectories collapsing onto a single path radically different from the forward trajectories (Label (c) in Fig. 5). Such a case is most common at topographical discontinuities or edges, such as coastlines. Finally, an intermediate *chaotic* regime (Label b in Fig. 5) appears primarily over land, where the trajectories diverge although the drift between their start and end points is limited. This behaviour is probably induced by orographic inhomogeneities that generate a complex climatic displacement pattern at the local scale.

### Trajectory regularity indices

**Figure 6:**
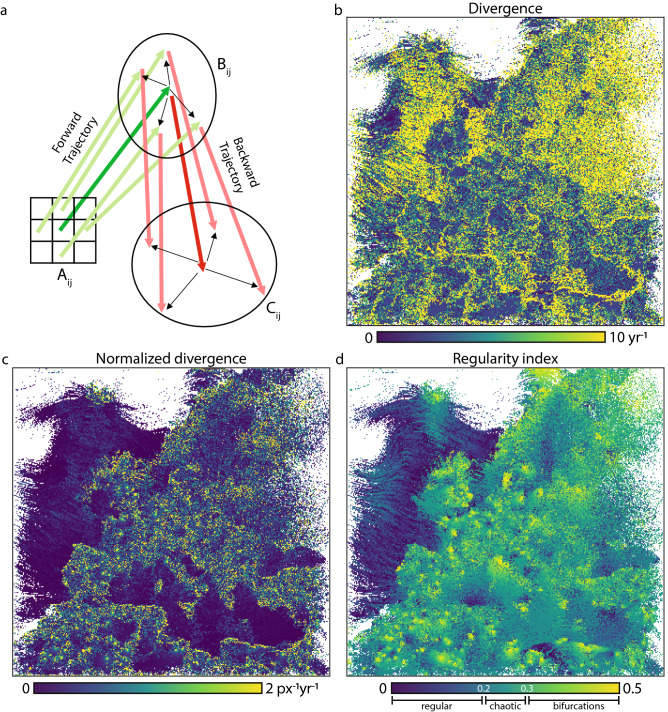
Quantification of the behaviours of forward-backward trajectory pairs (See main text for details). (**a**) Illustration of the nomenclature used for the definition of forward and backward trajectories. (**b**) Absolute value of the velocity field divergence. (**c**) Absolute value of the velocity field divergence, normalized by the length of the trajectory. (**d**) Regularity index, defined as half the Euclidean distance between the start and end point divided by the forward and backward trajectory endpoint distances (See Eq. (5)). The regularity index enables classification into regular, chaotic and bifurcation types of trajectories, with the latter closely related to isopleth sinks. Blank (white) points in all panels correspond to trajectories exiting the EUROCORDEX domain.

Beyond the qualitative typology of forward-backward trajectory pair behaviours, several metrics (Fig. 6) provide a more quantitative insight.

First, the horizontal divergence of the velocity field (Fig. 6b):

3 $$\begin{aligned} \overrightarrow{\nabla }\cdot \overrightarrow{v} = \frac{\partial v_x}{\partial x} + \frac{\partial v_y}{\partial y} \end{aligned}$$

$v_x$ and $v_y$ representing the zonal and meridional components of the horizontal wind vector, provides a generalization of Burrow’s^23^ convergence and divergence definition based on trajectory counting. It displays a clear contrast between blue regions where the absolute value of the divergence is below $\approx 5\hbox { yr}^{-1}$ and isotherm displacement is regular, and regions of high convergence or divergence (yellow regions of Figure 6b), where shearing of the velocity field is observed. The latter occurs both over land (North-Eastern plains) and over oceanic regions, especially close to coastlines and islands. The former corresponds to some open sea and oceans, as well as parts of the continental regions. While orography does not seem to play a big role in this case, this metric in fact is solely based on a the overall displacement map and does not take the full trajectory into account.

A better understanding is obtained when the divergence is normalised by the magnitude of the shifting velocity (Fig. 6b):

4 $$\begin{aligned} \overrightarrow{\nabla }\cdot {\overrightarrow{v}}_{norm} = \frac{\frac{\partial v_x}{\partial x} + \frac{\partial v_y}{\partial y}}{\overline{A_{ij}B_{ij}}}, \end{aligned}$$

$A_{ij}$ and $B_{ij}$ being the origin and the end of the trajectory issued from the initial grid point (*ij*), as depicted in Figure 6c. Here, the fast displacements over the ocean and over the North-Eastern plains result in low values, whereas local sinks and sources scattered over land show up clearly.

Finally, we define a *regularity index* (Fig. 6d) as:

5 $$\begin{aligned} {\mathscr {R}}_{ij}=\frac{1}{2}\frac{\overline{A_{ij}C_{ij}}}{ \overline{A_{ij}B_{ij}}+\overline{B_{ij}C_{ij}}} \end{aligned}$$

The regularity index corresponds to the ratio of the drift between the origin A$_{ij}$ and the backward trajectory endpoint C$_{ij}$, to the total excursion of the forward-backward trajectory (See Fig. 6a). As displayed in Fig. 6d, the high values of ${\mathscr {R}}$, *i*.*e*. chaotic trajectories, correspond to sinks, especially in coastal and mountainous regions, while the low values over the Atlantic correspond to smooth and parallel shifts. Although the normalized divergence (Fig. 6c) yields a comparable map, it provides less contrast. In fact, the regularity index can be seen as a generalization of the normalized divergence to forward-backward trajectory pairs.

## Discussion

We revisited the calculation of the climate shift velocity field by taking into account the mathematical requirement that, like any physical field, it should ultimately be continuous. This consideration, implemented within the proposed MATCH approach, allows us to calculate the shift velocity without any *ad hoc* assumption or approximation. In particular, it allows the shifting velocity to deviate from the gradient of the considered climate variable. This drastically improves the numerical stability and avoids locally infinite velocities as well as discontinuities of the velocity field. The resulting more continuous, less vortical, less noise-sensitive, and more detailed velocity maps, especially at the regional scale, allow a better analysis of local features, facilitate comparison between models, and allow us to define a trajectory regularity index to quantitatively assess sinks and sources during climate change.

Although we have mostly used temperature as an illustration, the continuity-based MATCH approach is applicable to any variable, whether scalar or vector, as long as it is defined and continuous throughout the region under consideration. This includes other climate variables such as humidity, precipitation, or wind, but also their statistical properties (including standard deviation, extreme values, and quantiles) or composite indices^25^. Similarly, the human impact of climate change can be investigated via the shifting velocity of climate-influenced parameters like agricultural productivity, harvest dates of crops of interest, or energy consumption for heating or air-conditioning buildings.

Beyond climate studies, the MATCH approach can be applied for shorter times and at various spatial scales to define a local velocity of climate change, as Loarie et al.^3^ did with the gradient-based method. For example, at the seasonal scale, it can be used to characterize the progression of vegetation blooming in spring and related herbivore migration (resource wave, or “Green wave”^26–28^). Specifically, the shift of isotherms or other isopleths may be related to the vegetation development front to help understand the displacement and migration trajectories^29^ of wild animals, although natural or man-made obstacles have to be considered as well. On the other hand, observational studies may help to assess the respective relevance of the gradient-, distance minimization-, and MATCH-based displacements for individuals, but also for populations where crowding effects may tend to regularize trajectories.

## Methods

### Continuity-based (MATCH) calculation of the shifting velocity fields

**Figure 7:**
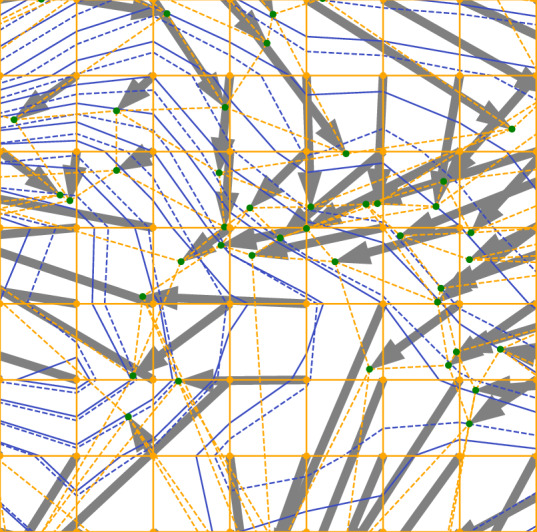
An example (See red square in Figure 2b for the actual location) of generating the local shifting velocity field using MATCH. In response to the displacement of the isotherms from solid blue to dashed blue conditions, the original regular orange grid deforms onto the dashed orange grid. This leads to a quantifiable displacement field with orange dots displacing into their new positions shown in green. The grey arrows display the vector field corresponding to these displacements.

We implemented the MATCH approach to the velocity field calculations as a Monte-Carlo-like^30^ iterative algorithm in Python, using standard scientific libraries. Here, for simplicity, we describe the method for the case of shifting isotherms, as depicted in Figure 7. However, this approach can be applied to any climate variable and its corresponding isopleths.

More specifically, we start from the initial regular rectangular grid (solid orange lines and dots) corresponding to the temperature map (solid blue lines display the isotherms) from the climate model. Grid points are displaced in a random sequence. When picked, each one is randomly displaced within the space bounded by nearest neighbors. By forbidding nearest-neighbour crossover, we trivially ensure the continuity condition. The temperature at this new position is interpolated from the final map, through the use of a fast bi-cubic spline algorithm^31^. If the move has reduced the norm of the difference between the initial and final temperatures, the displacement is retained, and the procedure is iterated with another randomly chosen grid point. Such an approach warrants an initial fast rough convergence followed by a gradual fine-tuning. After convergence is reached (*i*.*e*., when no further displacement reduces the difference between initial and final temperatures), the distorted grid (dashed orange lines in Fig. 7) maps the initial temperature values to a new location (green dots) in the final isotherm map (dashed blue lines). The resulting grid point displacement vectors (grey arrows) define a vector field that does not simply follow the direction of the temperature gradient. However, the MATCH continuity condition ensures that the displacement can be treated as a uniform shift - with neighbouring points starting and ending as nearest neighbors.

When needed for comparison, we also calculate the shifting velocity with the gradient-based method, in the standard way introduced by Loarie *et al.*^3^, following the implementation of Rey *et al.*^13^.

### Datasets

Global surface air temperature and pressure data simulated by the Canadian Centre for Climate Modelling and Analysis second generation Canadian earth system model (CanESM2 Global Climate Model^18,19^) were selected. We used daily values averaged over the periods 2006–2036 and 2071–2100 as the initial and final thirty-year periods, respectively, to estimate their shifts.

High-resolution (0.11°) simulated data is obtained from two regional climate model (RCM) outputs: HIRHAM5 (HIRAM, DMI^32^), and RACMO-22E (RACMO, KNMI^33^) over the 1951–2100 period, within the EURO-CORDEX initiative^34,35^. The computational domain covers the following geographical coordinates: (63.55° N, 51.56° W), (63.65° N, 72.56° E), (20.98° N, 14.31° W), and (20.98° N, 72.56° E). The ability of EURO-CORDEX RCM simulations to reproduce contemporary temperature statistics has been previously reported^36,37^ and the outputs have been widely used to analyze projections of temperatures in Europe^38^.

In this work, we focus on the high-emission RCP8.5 scenario, in which the average surface warming is projected to reach 4° by the end of the XXIst Century relative to the 1986–2005 average^16^. Simulated temperature from either models are averaged over 30-years periods and used to compute shifting velocity fields from 1950–1980 to 1980–2010, as well as from 2040–2070 to 2070–2100.

### Trajectories and back trajectories

Isotherm shifts were computed using annual mean screen-level air temperature simulated with Regional Climate Models (RCMs), whereby year-to-year fluctuations were averaged out at each grid point by a third order polynomial fit. MATCH was then applied for each pair of successive years. The resulting sequences of yearly shifts was integrated into full-length trajectories after another bicubic spline interpolation to reduce the effects of inter-annual variations.

Back trajectories were calculated using the same methodology, but applied to the time-inverted temperature series. We note that the presence of *sources* and *sinks* does not allow a unique back trajectory to be computed through matrix inversion methods.

Finally, boundary conditions were set to terminate trajectory calculation in the event it went out of the data bounds. In this case, the trajectory was flagged as invalid and disregarded in the subsequent quantitative analysis.

### Datasets

The datasets analysed during the current study are available in^34,35^ (Euro-Cordex dataset) and^18^ (CCCma). The Python application used to explore the different behaviours and to generate Fig. 5 is available online^24^.

## Supplementary Information

Supplementary Information.
