# Supplementary material for: Smooth velocity fields for tracking climate change
Source: Sci Rep. 2022 Feb 22;12:2997. doi: 10.1038/s41598-022-07056-z (PMC8863831; doi:10.1038/s41598-022-07056-z)
Supplement: Supplementary file 1 — Supplementary Information. [file 41598_2022_7056_MOESM1_ESM.pdf]

Supplementary Material: Smooth velocity fields for tracking climate change

Iaroslav Gaponenko[1], Guillaume Rohat †[2], Stéphane Goyette[2,3], Patrycja Paruch[1], and Jérôme Kasparian[2,3,*]

[1]DQMP, University of Geneva, Quai Ansermet 24, 1211 Geneva 4, Switzerland
[2]Institute for Environmental Sciences, University of Geneva, bd Carl Vogt 66, 1211 Geneva 4, Switzerland
[3]Group of Applied Physics, University of Geneva, Chemin de Pinchat 22, 1211 Geneva 4, Switzerland
[*]jerome.kasparian@unige.ch

ABSTRACT

This Supplementary Material present supporting information and figures for the main text.

Projection of displacements onto a sphere

Both the output from the models as well as the computations performed with the MATCH/GB methods were done on a grid with equal latitude/longitude spacing. In order to provide for a real-space representation, the displacements shown in Figure 2 have been mapped onto 3D spheres as shown in Figure S1. Additionally, animations were generated with a rotating view and are available as GIF files in the Supplemental Materials and at the Yareta repository[1].

CCCma CanESM2 RCP 8.5 isobar displacement

The shifting velocity of mean sea-level pressure computed with the MATCH method (Figure S2b) evidences the displacements of extra-tropical storm tracks in the Northern Hemisphere, primarily over the Atlantic and Pacific Oceans. In the Southern Hemisphere, these tracks are more zonally uniform. As climate warms, storm tracks are indeed predicted to shift poleward[2]. With the gradient-based method (Figure S2a), these features are only visible over land, while over the ocean this approach results in shifting velocities close to zero.

Zonal statistics

Azimutal angle maps and their zonal-averaged meridional profiles have been computed for the global dataset presented in Figure 2.The resulting maps and profiles are shown in in Figure S3. It is worth noting that the MATCH method has a finer level of detail than the gradient-based (GB) approximation, especially within the coastline and land surfaces where the GB method breaks down. Indeed, as can be seen by comparing Figure S3a with Figure S3b, the GB method gives a more artificial result that is correlated with the presence of topographic features. This is due to the GB method only considering the spatial gradient of the initial isopleth and the temporal evolution thereof - whereas the MATCH method takes into account the initial and final positions of the isopleths, generating a true trajectory.

GB vs MATCH displacement correlation

To estimate the similarity between the MATCH and GB methods, a two-dimensional correlation histogram was plotted in Figure S4. Globally, a unity correlation is observed with a non-trivial spread. At low displacements, such as in the case of Figure S4(a), a small asymmetry appears, with the Gradient-based method overestimating the former. In the higher displacements regime such as in Figure S4(b), the Gradient-based method tends to underestimate the displacements.

Additional online information

The following are available in the Supplemental Materials and at the Yareta repository[1]:

- GIF format rotating 3D sphere renderings as shown in Figure S1;
- Python code and example data for the MATCH method, built into an ipython notebook.

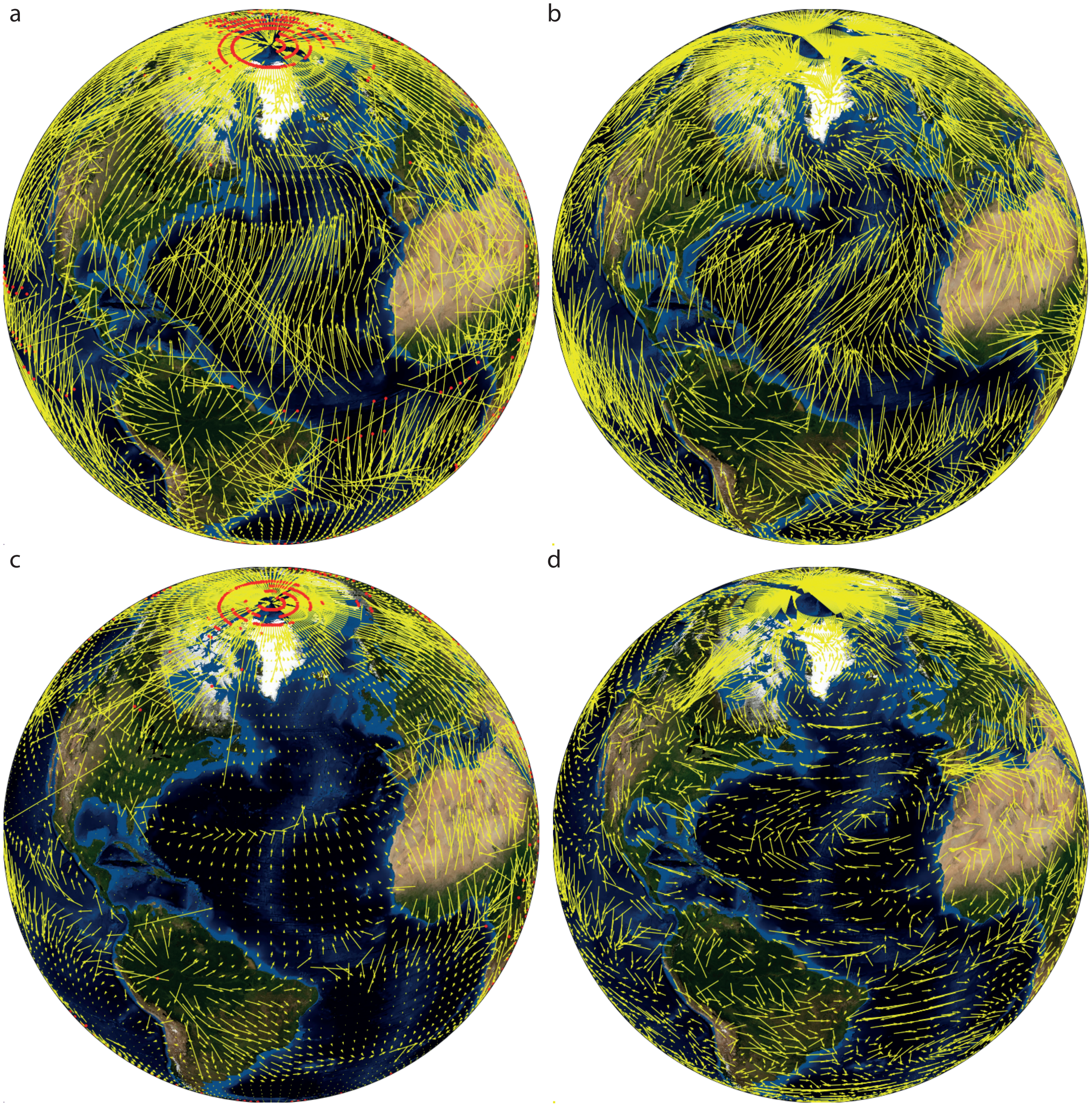

Figure S1. Shifting isotherms (a,b) and isobars (c,d) between periods 2006 – 2036 and 2070 – 2100 as simulated by the CCCma CanESM2 for the RCP 8.5 scenario, projected onto 3D spheres. (a,c) Gradient approach; (b,d) MATCH. Red dots in the gradient approximation represent displacement beyond 40 pixels apart (given 2.82° per pixel), which were removed from consideration due to their nonphysical signature. This figure has been produced using the code available in the Additional online information (See Supplementary Materials), using the Anaconda distribution of Python 3.6.8, with matplotlib 3.1.1 and numpy 1.14.3, available at https://www.anaconda.com/

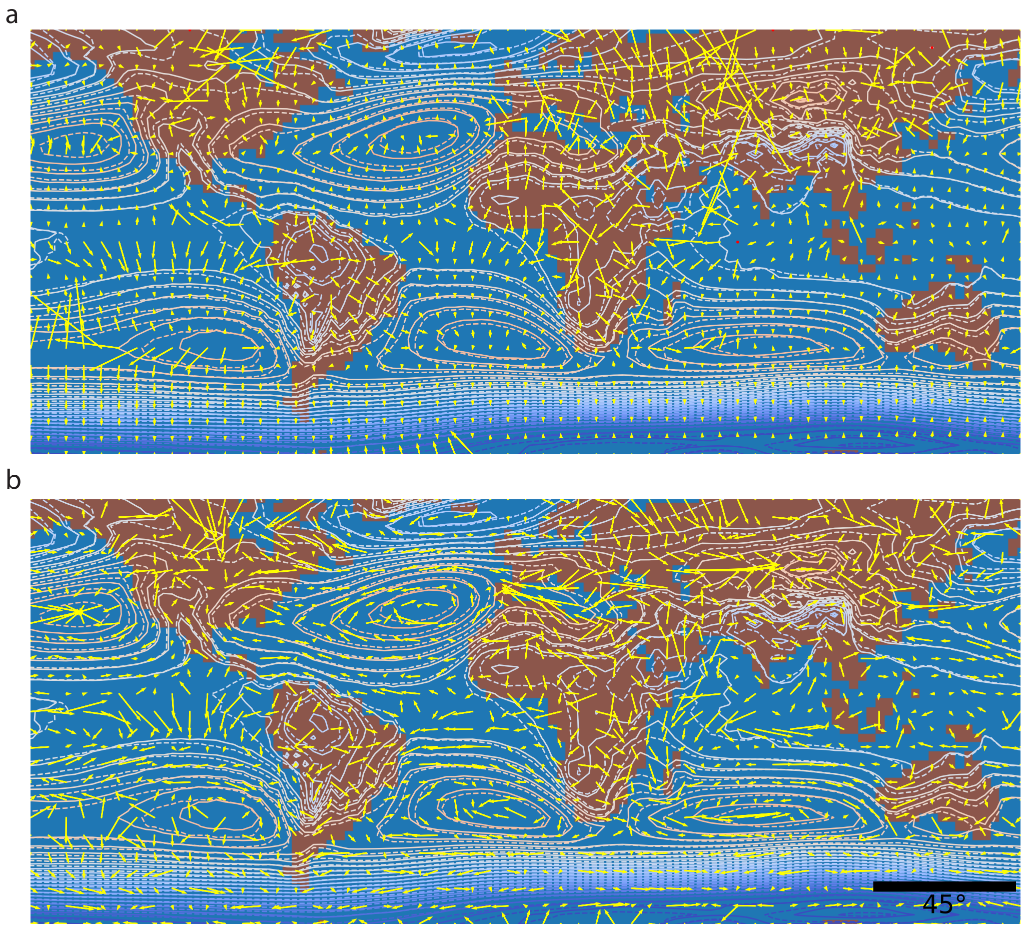

Figure S2. Shifting velocity field of isobars between periods 2006 – 2036 and 2070 – 2100. The isobars, for the (a) gradient and (b) MATCH methods are taken from the CCCma CanESM2 model for the RCP 8.5 scenario, with the same considerations as Main Figure 2. This figure has been produced using the code available in the Additional online information (See Supplementary Materials), using the Anaconda distribution of Python 3.6.8, with matplotlib 3.1.1 and numpy 1.14.3, available at https://www.anaconda.com/

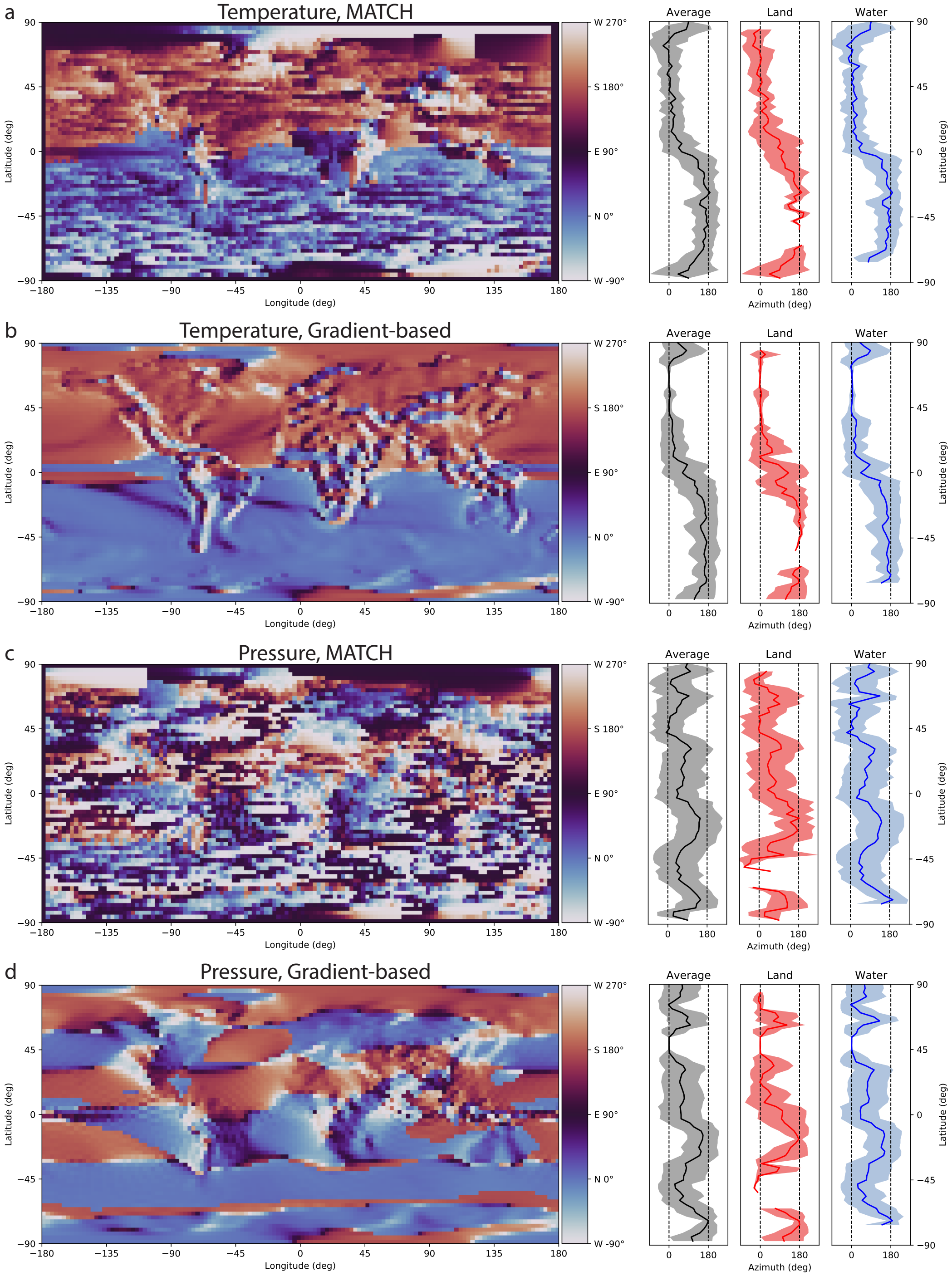

Figure S3. Global azimutal angle maps and zonal-averaged meridional profiles thereof (solid lines) for global (black), overland (red) and over oceans areas (blue), as well as standard deviations (shaded areas) for the 2-m air temperature and mean-sea-level pressure analysed in the main text, according to the MATCH (a,c) and Gradient-based (b,d) methods. Shifting azimuts pointing to the North (0°), East (90°), South (180°), and West (−90°) and latitudes range from the Equator (0°) to the North (90°) and South Poles (−90°. Also the data gaps in the southern hemisphere azimutal shifts over oceans indicate that there is no continental surfaces within these zones.) This figure has been produced using the code available in the Additional online information (See Supplementary Materials), using the Anaconda distribution of Python 3.6.8, with matplotlib 3.1.1 and numpy 1.14.3, available at https://www.anaconda.com/

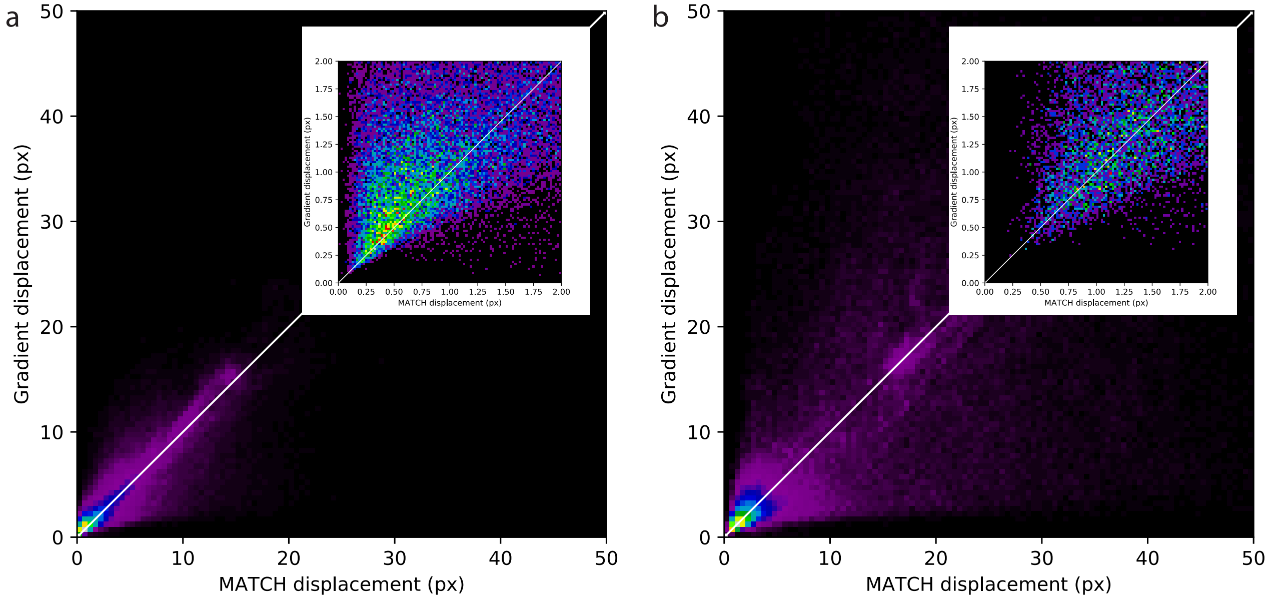

Figure S4. Two-dimensional histogram maps of Gradient-based vs MATCH shifts for the regional displacement fields for the surface temperature under the RCP8.5 scenario as computed by the RACMO model for two time periods: (a) 1950 – 1980 to 1980 – 2010 and (b) 2040 – 2070 to 2070 – 2100. Globally, there is a clear correlation along the white $y = x$ line. It is worth noting that for the lower displacements in (a) the Gradient-based method overestimates the displacements, whereas it underestimates them in the high displacement regime in (b).

- Python code and data for the visualization of the individual trajectories described in the *Isotherm trajectories* section of the main paper.

References

1. Yareta repository with supporting data and code, DOI: 10.26037/yareta:nhslc5mgrjeclgqwzuvri6lgkq (2020).
2. Bengtsson, L., Hodges, K. I. & Roeckner, E. Storm tracks and climate change. *J. Clim.* **19**, 3518–3543, DOI: 10.1175/jcli3815.1 (2006).
